# Phytochemical-Stabilized Platinum-Decorated Silver Nanocubes INHIBIT Adenocarcinoma Cells and Enhance Antioxidant Effects by Promoting Apoptosis via Cell Cycle Arrest

**DOI:** 10.3390/pharmaceutics14112541

**Published:** 2022-11-21

**Authors:** Adewale Odunayo Oladipo, Jeremiah Oshiomame Unuofin, Sogolo Lucky Lebelo, Titus Alfred Makudali Msagati

**Affiliations:** 1 Department of Life and Consumer Sciences, College of Agriculture and Environmental Sciences, University of South Africa, Private Bag X06, Florida, Johannesburg 1710, South Africa; 2 Institute for Nanotechnology and Water Sustainability (iNanoWS), College of Science, Engineering and Technology, University of South Africa, Private Bag X06, Florida, Johannesburg 1710, South Africa

**Keywords:** antioxidant, anticancer, silver-platinum nanoparticles, apoptosis, cell cycle arrest, cell-specific response

## Abstract

(1) Background: The increasing use of silver and platinum bimetallic nanoparticles in the diagnosis and treatment of cancer presents significant advances in biomedical applications due to their extraordinary physicochemical properties. This study investigated the role of aqueous phytochemical extract in stabilizing platinum nanodots-decorated silver nanocubes (*w*-Pt@AgNPs) for enhancing antioxidant activities and their mechanism. (2) Methods: UV-Vis, Fourier transform infrared (FTIR) spectroscopy, and transmission electron microscopy (TEM) were used to characterize the formed *w*-Pt@AgNPs. LC-QToF-MS/MS was used to analyze the bioactive compounds, while DPPH, ABTS, and FRAP were used to detect the scavenging potential. Flow cytometric assays were performed to investigate the cytotoxicity and the mechanism of cell death. (3) Results: Morphological studies indicated that *w*-Pt@AgNPs were cube in shape, decorated by platinum nanodots on the surfaces. Compared to ethanolic extract-synthesized *e*-Pt@AgNPs, *w*-Pt@AgNPs exhibited the strongest antioxidant and cytotoxic activity, as data from Annexin V and Dead cell labeling indicated higher induction of apoptosis. Despite the high proportion of early apoptotic cells, the *w*-Pt@AgNPs triggered a decrease in G1/G0 cell cycle phase distribution, thereby initiating a G2/M arrest. (4) Conclusions: By enhancing the antioxidant properties and promoting apoptosis, *w*-Pt@AgNPs exhibited remarkable potential for improved cancer therapy outcomes.

## 1. Introduction

Recently, green nanotechnology has rapidly gained immense attention as an alternative method of fabricating multimetallic nanoparticles [1,2,3]. The fabrication of multimetallic nanoparticles via eco-friendly approaches has increasingly resulted in the synthesis of stable, cheaper, and biocompatible nanoparticles, thus limiting the deleterious effects of using toxic chemicals in their synthesis [4,5]. With a significant broad area of application in environmental [6], industrial [7], and biomedical science [8], multimetallic nanoparticles are increasingly studied due to their enhanced optical [9], electronic [10], catalytic [11], and synergistic properties [12,13] which differ greatly from that of the individual monometallic nanoparticles [14].

Among nanostructures with multi-metal constituents, bimetallic nanoparticles, formed by combining two distinct metallic nanoparticles into a single entity have provoked scientific and technological interests. The architectural mixing patterns generate an extra degree of freedom which enables the optimization of the surface plasmon absorption of the individual metal, thus, leading to enhanced functionalities [15,16]. By merely changing an individual metal component or ratio, the physicochemical properties can be modified due to the synergistic effects between the metals [17,18]. Through the bimetallization process, resulting nanoparticles have emerged with superior stability, selectivity, and therapeutic efficiency compared to monometallic nanoparticles [19]. While diverse bimetallic metal combinations such as Au-Pt [20], Au-Ag [3], Au-Pd [21], Pd-Pt [22], Ag-Pt [23], Pt-Cu [24], etc. have been reported, Ag-Pt stands out as a fascinating combination. In most cases, the Ag-Pt bimetallic nanoparticles combine the excellent antimicrobial properties of Ag with the cytotoxic potency of Pt in a synergistic approach. As for Ag-Pt bimetallic nanoparticles, several studies have reported the following special characteristics: (a) enhanced catalytic and antimicrobial activities [25,26], (b) shape and size-induced higher tunable near-infrared (NIR) absorption [27,28], (c) higher surface-enhanced Raman scattering (SERS) activities [29,30], and (d) improved cytotoxic potency against cancer cells [31,32] relative to their single metal counterparts. These fascinating properties have found interesting potential in many biomedical applications, such as photothermal and photodynamic therapies for cancer [33], antimicrobial [34], and multidrug resistance treatment [35] due to their better functionalities. Despite these intriguing properties, their effectiveness depends on their shape, size, composition, and atomic arrangement within the nanoparticles. Hence, it is desirable to fabricate bimetallic nanoparticles with unique structural properties for optimal efficiency.

In previous studies on the biosynthesis of Ag-Pt nanoparticles, a large portion of these nanoparticles has been observed to have mostly core-shell and alloy in geometrical orientation. This has been attributed to the broad miscibility gap between silver and platinum [32]. For example, bimetallic silver-platinum nanoparticles with alloyed orientation were demonstrated to combine antimicrobial activity with osteo-promotive properties on human mesenchymal stem cells [26]. Similarly, silver-platinum nano assembly with dendritic structures showed significant inhibitory responses toward microbial and cancer cells [31]. In another report, core-shell Ag-Pt bimetallic nanoparticles showed cytotoxic potential towards human mesenchymal stem cells due to the release of only Ag^+^, whereas hollow-shaped Ag-Pt with platinum-rich particles did not result in Ag^+^ release [32]. Recently, our group reported the green synthesis of AgPt alloyed nanoparticles with potential antioxidant, antimicrobial and cytotoxic activities [36]. These studies have demonstrated the dependence of the various applications of Ag-Pt bimetallic nanoparticles on their morphology/structural orientations and remain a continuous subject of research.

A promising alternative eco-friendly method uses an extract from plants with medicinal properties. Medicinal plants, due to their rapid and efficient formation mechanism, biocompatibility, and the ability of their phytochemicals to interact strongly with the formed nanoparticles, have become preferred over other biogenic methods [22,37]. *Vernonia mespilifolia* is a medicinal plant endemic to Southern Africa [38]. Numerous curative capabilities, such as heartwater disease treatment for ruminant animals, high blood pressure, and body weight management in humans, to name a few are the potential of *V. mespilifolia* [39,40]. In addition, the extracts from *V. mespilifolia* are predominantly rich in phytochemicals such as polyphenols, tannins, saponins, and flavonoids [41] which we hypothesized could provide a natural reducing and stabilizing platform for the synthesis of bimetallic nanoparticles.

Therefore, the present study was designed for the eco-friendly synthesis of Pt@AgNPs using ethanolic and aqueous *V. mespilifolia* extracts and the characterization of the synthesized nanoparticles utilizing solid-state techniques. The effect of the type of solvent used for phytochemical extract in the formation of the nanoparticles and the properties of the obtained Pt@AgNPs were evaluated to direct these properties for potential biomedical applications. In addition,, the in vitro antioxidant, anticancer properties, and the mechanism of cell death induced by the synthesized nanoparticles were investigated.

## 2. Materials and Methods

### 2.1. Materials and Reagents

All the chemicals used in this study were purchased from Merck, Johannesburg, South Africa, and used as obtained. The whole *V. mespilifolia* plant was collected from the wild (latitude 32° 51′41.846″ S and longitude 27°10′59.318″ E) in the Eastern Cape province, South Africa. All precursor solutions were prepared in deionized water.

### 2.2. Preparation of Plant Material and Aqueous Extract

The whole plant powder was prepared as earlier reported [41]. One gram of plant powder was dispersed in 100 mL of deionized water and heated for 20 min at 60 °C and then filtered using Whatman filter paper. The obtained extract was stored at 4 °C before nanoparticle synthesis.

### 2.3. Synthesis of w-Pt@AgNPs

For the platinum-decorated silver nanoparticles (*w*-Pt@AgNPs), an aqueous extract (10 mL) of *V. mespilifolia* (*w*VM) was mixed with a 50 mL solution of (1 mM AgNO_3_ and 1 mM K_2_PtCl_4_, Merck, Johannesburg, South Africa) in a flask at room temperature and stirred continuously at 85 °C for 1 h. The reaction was carried out in the dark to avoid unnecessary light interactions. The obtained nanoparticles were centrifuged several times (4400 rpm for 30 min) with water to remove any unreacted salts and extracts. The pellets obtained were then oven-dried at 40 °C to collect the nanoparticles in powder. A similar procedure was followed to synthesize *e*-Pt@AgNPs from an ethanolic extract (*e*VM) of *V. mespilifolia*.

### 2.4. LC-QToF-MS/MS Analysis

Metabolites present in the *w*VM were investigated using LC-QToF-MS/MS in the positive mode of ionization. Dried extract (1 mg) was dissolved in 1 mL of LC-MS grade water and sonicated for 10 min. The solution was filtered using a 0.22 mm polyvinylidene fluoride (PVDF) membrane syringe filter, and the obtained sample was analyzed using a high-resolution Impact II Quadrupole-time of flight mass spectrometer (QToF-MS) (Bruker, Bremen, Germany). Data analysis was conducted using Compass Data Analysis software v4.3 (Bruker Daltonics, Germany), and the MetFrag web tool was used to compare the fragment patterns of ions obtained with those from KEGG and ChEBI databases.

### 2.5. Characterization of the Synthesized Pt@AgNPs

Fourier-Transform Infrared spectroscopy (FTIR) analysis was performed using a PerkinElmer (Frontier FT-IR) fitted with an ATR detector. The bioactive compounds present in the *w*VM and *e*VM extracts, as well as the reduced *e*-Pt@AgNPs and *w*-Pt@AgNPs, were recorded in the range of 500–4000 cm^−1^. The vibrational frequencies obtained from the spectra analysis were used to determine the various functional groups present in the extracts and those associated with the nanoparticles. The morphology and particle size of the Pt@AgNPs were determined by Transmission Electron Microscopy (TEM) on a JEOL JEM 2100 instrument running at 200 kV voltage. The dried samples were dispersed in water and agitated for 10 min before the samples were dropped on a carbon-coated copper grid thin film. The copper grid was allowed to dry before the investigation of the shape of the Pt@AgNPs. Energy-dispersive X-ray spectra (EDS) were measured on a TEM equipped with an energy-dispersive X-ray spectrometer. Dynamic Light Scattering (DLS) technique on a Malvern Zetasizer (Nano-ZS, Malvern, UK) was used to assess the hydrodynamic diameter, polydispersity index (PDI), and stability of the nanoparticles.

### 2.6. Total Phenolic, Flavonoid, and Proanthocyanidins Content Estimation

The total phenolics, flavonoids, and proanthocyanidins present in the nanoparticles were estimated according to the methods reported [41,42]. The contents were expressed in milligram gallic acid (for phenolic content) or quercetin (for flavonoid content), or catechin (for proanthocyanidins content), the equivalent per gram of extract.

### 2.7. Antioxidant Activity

Three antioxidant assays {(ABTS (2,2′-azino-bis (3-ethylbenzothiazoline)-6-sulfonic acid), DPPH (2,2-diphenyl-1-picrylhydrazyl), and ferric reducing antioxidant power (FRAP)} were used to determine radical scavenging capacities of *w*-Pt@AgNPs and *e*-Pt@AgNPs. Antioxidant assays were performed according to methods adopted from a previous study [36]. 

### 2.8. Cell Cultures

Human embryonic kidney (HEK 293), human breast carcinoma (MCF-7), and human lung carcinoma (A549) cell lines were obtained from the cell bank of Fly Lab, College of Agriculture and Environmental Sciences (CAES), University of South Africa. Cells were cultured in Dulbecco’s Modified Eagle’s medium (DMEM), Merck, Johannesburg, South Africa) which were supplemented with fetal bovine serum (10% FBS, Merck, Johannesburg, South Africa), 1% Glutamine (Gln), and 1% Penicillin–streptomycin (Merck, Johannesburg, South Africa).

#### 2.8.1. Cell Proliferation Assay

The cell proliferation assays were conducted to evaluate the cytotoxicity of the as-synthesized nanoparticles on HEK 293, MCF-7, and A549 cells following the fluorometric assay using CellTiter-Blue reagent (Promega, Madison, WI, USA) according to the manufacturer’s guide. Cells were seeded into a 96-well plate at 2 × 10^4^ cell/well at 50 µL/well, and the plates were incubated for 24 h at 37 °C. After incubation, the cells were exposed to 50 µL of various concentrations of *w*-Pt@AgNPs and *e*-Pt@AgNPs dispersed in culture media and allowed to culture for 24 h and 48 h. At the end of the incubation time, 20 µL/well of CellTiter Blue reagents were added to each well. The plates were shaken for 10 s and further incubated for 4 h. Fluorescence values were recorded at 560 nm (excitation) and 590 nm (emission) using an ELISA microplate reader (ThermoFisher Scientific, Varioskan Flash, Vantaar, Finland), and the cell viability of the triplicate results estimated.

#### 2.8.2. Annexin V and Dead Cell Apoptosis Assay Using Flow Cytometry

Human breast carcinoma (MCF-7) and human lung carcinoma (A549) cells were assessed for the induction of apoptosis using the GUAVA MUSE Cell Analyzer (Luminex Corp., Austin, TX, USA). Briefly, after incubation for 24 h at a concentration of 1 × 10^5^ cells/mL in a 6-well plate, the cells were treated with *w*-Pt@AgNPs for 24 h. Following this, the cells were harvested with trypsin-EDTA and washed twice with PBS to remove nanoparticles not taken up by the cells. Thereafter, the cells were centrifuged and resuspended inside a tube with fresh 100 µL PBS under gentle vortexing. 100 µL of Annexin V & Dead Cell reagent was added to the resuspended cells and incubated at room temperature in the dark for 20 min. The percentage of apoptotic and dead cells was analyzed using a flow cytometry fitted with a Muse Cell Analyzer software module system. The experiments were performed in triplicate, and the results were expressed as the percentage of apoptotic cells and standard deviation. The untreated cells were used as a negative control, while cells treated with *e*-Pt@AgNPs were used as positive control.

#### 2.8.3. Cell Cycle Arrest Using Flow Cytometry

Human breast carcinoma (MCF-7) and human lung carcinoma (A549) cells were seeded at 1 × 10^5^ cells/mL in a 6-well plate and incubated for 24 h at 37 °C. After treatment with *w*-Pt@AgNPs and *e*-Pt@AgNPs for 24 h, cells were transferred into a 1.5 mL conical tube and centrifuged at 1500 rpm for 5 min. Thereafter, the cells were washed once with PBS, and the concentration was adjusted to 1 × 10^6^ cells/mL before fixing for 3 h with ice-cold 70% ethanol at −20 °C. Samples were centrifuged and washed once with PBS, and the pellets were resuspended in 200 µL PBS inside a 1.5 mL tube, followed by the addition of a 200 µL MUSE Cell Cycle reagent. The tube containing a mixture of cells and reagents was incubated for 30 min in the dark at room temperature before analysis using the GUAVA MUSE Cell Analyzer (Luminex Corp., Austin, TX, USA) equipped with an analyzer software module system. The percentage of cells in G0/G1, S, and G2/M phases and debris were calculated by the software.

### 2.9. Statistical Analysis

All experiments were performed in triplicate, and all the data were expressed as mean ± standard deviation (SD). Statistical analysis was performed using MINITAB 17 and student *t*-test followed by the Wilcoxon test for comparison. The difference between the mean values of groups was considered to be significant at * *p* < 0.05 and ** *p* < 0.01.

## 3. Results

### 3.1. Phytochemical Profiling of Aqueous Plant Extract by LC-QToF-MS/MS

**Figure 1:**
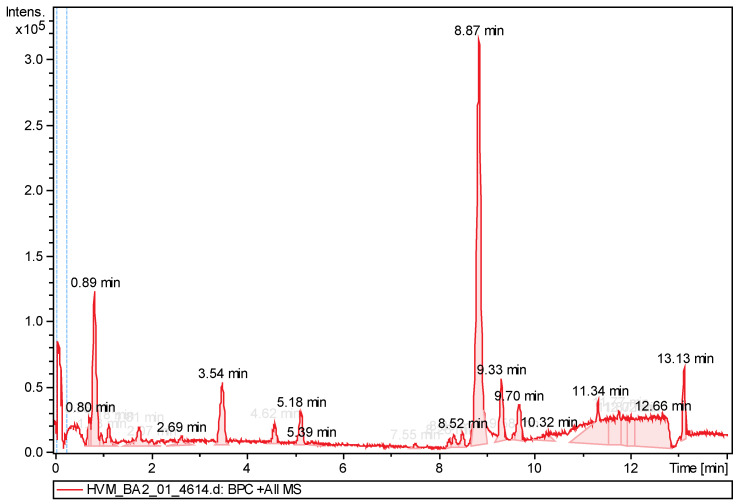
Base peak chromatogram (BPC) of the aqueous extract from *w*VM.

Phytochemical screening of the aqueous extract of *V. mespilifolia* (*w*VM) revealed the presence of phytocompounds with highly reactive functional groups. Figure 1 shows the chromatogram of *w*VM, while key compounds identified with their corresponding retention time (RT), molecular weight, molecular formula, and structures are shown in Table 1. The LC-QToF-MS/MS analysis carried out on *w*VM showed the presence of the following metabolites: proline, N6-methyl-2″-deoxyadenosine, 4-guanidino-1-butanol, 4-guanidinobutanoate, phenylpyruvate, bis-D-fructose2″,1:2,1″-dianhydride, epsilon-caprolactam, 2-hydroxy-7-hydroxymethylchromene-2-carboxylate, and vigabatrin. These compounds have been documented to possess antioxidant, antibacterial, anti-inflammatory, antidiabetic, and other pharmacological activities. Among the identified compounds, proline has demonstrated reactive oxygen species scavenging properties [43], while phenylpyruvate, under natural UV light, has been shown to generate free radicals, resulting in the induction of DNA damage [44].

### 3.2. Characterization of Platinum-Silver Nanoparticles (w-Pt@AgNPs)

#### 3.2.1. Spectroscopic Analysis

**Scheme 1:**
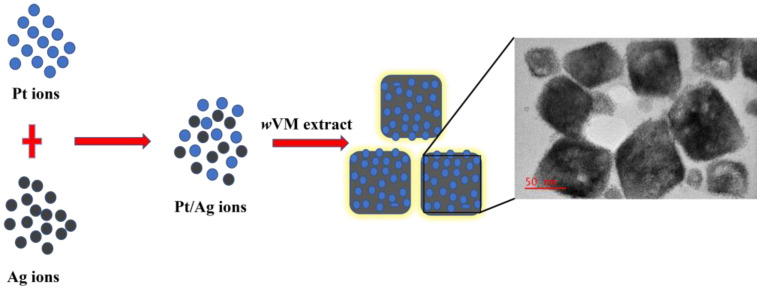
An illustration of the proposed mechanism of formation of *w*-Pt@AgNPs using the *w*VM extract.

**Figure 2:**
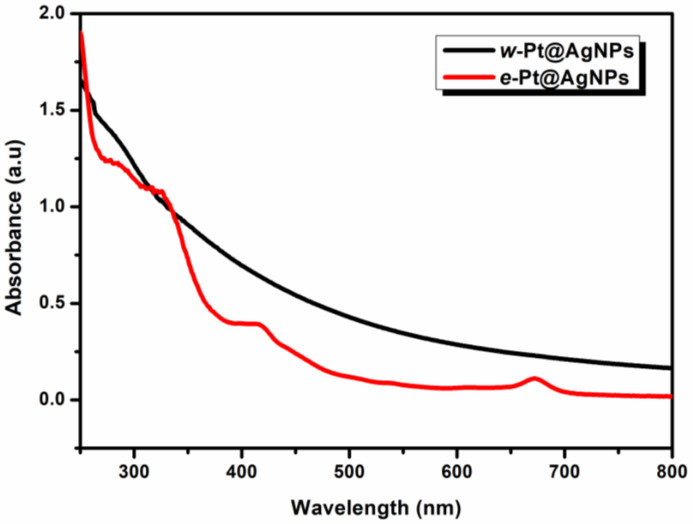
UV-vis absorption spectra of extract-synthesized, *e*-Pt@AgNPs and *w*-Pt@AgNPs.

UV-Vis spectroscopy, an important technique, was used to investigate the formation of both the *e*-Pt@AgNPs and *w*-Pt@AgNPs (Scheme 1). The formation of *w*-Pt@AgNPs showed a broad absorption band stretching toward the near-infrared region of the spectrum (Figure 2). However, the ethanolic extract-synthesized *e*-Pt@AgNPs showed a single plasmon absorption band at 414 nm, characteristic of Ag nanoparticles [45]. The typical Pt (II) peak of 262 nm disappeared from this spectrum and was replaced by a peak at 327 nm, suggesting that different sizes and shapes of bimetallic nanoparticles were formed. The band at 291 nm and 673 nm noticed in the *e*-Pt@AgNPs spectrum could be attributed to the strong interaction of the nanoparticles with the phytochemicals from the *w*VM extract. Unlike alloyed and core-shell nanoparticles, which display single and double absorption bands, respectively [20], the disappearance of the absorption band in the *w*-Pt@AgNPs demonstrated the sensitivity of the surface plasmon resonance (SPR) absorption to additional metal incorporation, thereby indicating a possible coverage of the AgNPs surface by Pt nanodots [46].

#### 3.2.2. Morphological Analysis

**Figure 3:**
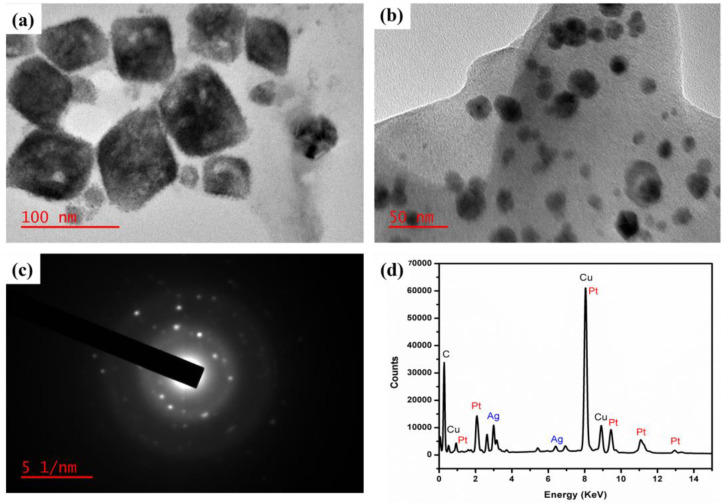
TEM images showing the morphology of (**a**) *w*-Pt@AgNPs and (**b**) *e*-Pt@AgNPs, SAED (**c**), and EDS spectra (**d**) of *w*-Pt@AgNPs.

The structural architecture of the *w*-Pt@AgNPs was explored by transmission electron microscopy (TEM) and electron diffraction technique. The TEM image of the biosynthesized *w*-Pt@AgNPs revealed a cube-shaped Pt@AgNPs (Figure 3a). The cubic-shaped Pt@AgNPs were decorated by ultrasmall-sized platinum nanodots all around the surfaces. In contrast, the *e*-Pt@AgNPs formed from the *e*VM extract were predominantly spherical (Figure 3b). As shown in Figure S1, the particle size distribution (~ about 65 nanoparticles measured) of both *w*-Pt@AgNPs and *e*-Pt@AgNPs were 75.3 ± 0.4 nm and 35.7 ± 1.2 nm in diameter, respectively The selected area electron diffraction (SAED) pattern of the *w*-Pt@AgNPs showed several diffraction rings in the pattern with bright spots around a single nanoparticle (Figure 3c). The ring patterns can be assigned to (111), (200), (220), and (311) planes of Ag-Pt nanoparticles and indicate a face-centered cubic (*fcc*) structure, thus, confirming the polycrystalline nature of the biosynthesized *w*-Pt@AgNPs. A similar electron diffraction pattern with bright spots was shown by the *e*-Pt@AgNPs (Figure S2). The elemental composition of the nanocubes was characterized by energy-dispersive X-ray (EDX) spectroscopy. Figure 3d shows the EDX spectra of the *w*-Pt@AgNPs, where Ag and Pt peaks present with the carbon and copper peaks coming from the carbon-coated copper grid used for the analysis. Similarly, the *e*-Pt@AgNPs displayed corresponding Ag and Pt peaks from the EDX spectra in Figure S3. Both *w*-Pt@AgNPs and *e*-Pt@AgNPs are in the nanoscale range (below 100 nm), which could be easily internalized by cells.

#### 3.2.3. FTIR Analysis

**Figure 4:**
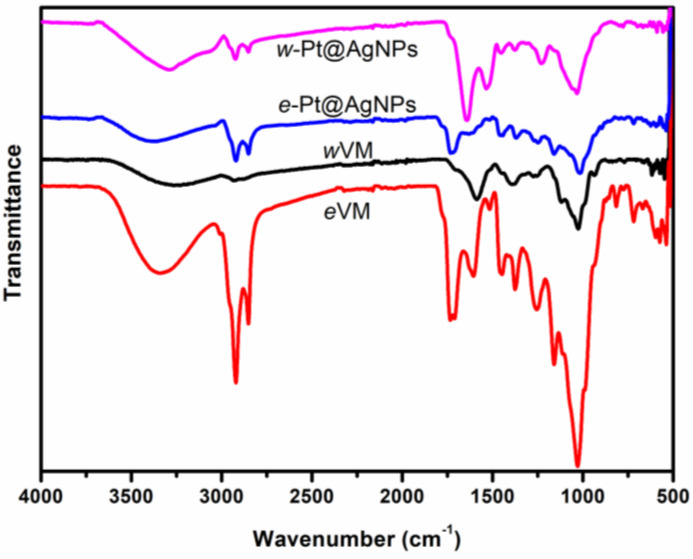
Fourier transform infrared spectra of ethanolic and aqueous VM extracts and their corresponding Pt@AgNPs.

FTIR technique was used to evaluate the functional groups present in the two extracts and their possible interaction during the formation of the nanoparticles. The FTIR spectra of the dry powdered extract from *w*VM and *e*VM, as well as the synthesized *w*-Pt@AgNPs and *e*-Pt@AgNPs, are shown in Figure 4. Distinguishing peaks of *w*VM were observed at 3255, 2932, 2887, 1582, 1400, and 1026 cm^−1^. The peaks at 3255 cm^−1^, 1582 cm^−1^, 1400 cm^−1^, and a strong, intense peak at 1026 cm^−1^ corresponded to the -OH stretching vibrations (alcohols and phenols); C=O stretching vibrations (flavonoids); C-H stretching vibrations (aromatic and aliphatic amines); and C-N stretching vibrations (amines) respectively. The characteristic bands that appeared in both *w*VM and *e*VM could mostly be detected in the spectra of extract-formed *w*-Pt@AgNPs and *e*-Pt@AgNPs. For example, the spectra of *w*-Pt@AgNPs and *e*-Pt@AgNPs showed bands at 3295 cm^−1^ and 3372 cm^−1,^ respectively, corresponding to the O-H bands. The peaks at 2924 cm^−1^ and 2856 cm^−1^ (asymmetric C-H bands; alkanes) found in the *e*VM extract were also visible in the *e*-Pt@AgNPs spectra. On the contrary, these C-H bands were more pronounced in the *w*-Pt@AgNPs formed than in the *w*VM alone. The intense peaks at 1734 cm^−1^ and 1023 cm^−1^ in the *e*VM became broad after the formation of the *e*-Pt@AgNPs. Several intense peaks in *e*VM spectra were reduced in intensity in the spectrum of *e*-Pt@AgNPs. Additionally, new peaks (1650 and 1527 cm^−1^) could be seen in the spectra of the *w*-Pt@AgNPs after its formation, while the 1033 cm^−1^ and 1024 cm^−1^ observed in both Pt@AgNPs were due to C-N stretching vibrations of amine. These results confirmed that the phytochemicals present in the extracts had a strong tendency to bind onto metals, thereby mitigating the possibility of aggregation to occur. Similarly, the functional groups of the extracts contained phenols, flavonoids, and tannins which provided not only efficient capping but also involved in the reduction and stabilization of the nanoparticles.

#### 3.2.4. Stability Studies

The zeta potential is regarded as a crucial feature in materials characterization due to its strong relationship with the stability of colloidal nanoparticles. According to Sharma et al. [47], we dispersed *w*-Pt@AgNPs and *e*-Pt@AgNPs in deionized water and assessed the zeta potential. For *w*-Pt@AgNPs and *e*-Pt@AgNPs, an average value of −30.3 ± 2.81 mV and −25.0 ± 2.11 mV was calculated, respectively. Furthermore, the zeta potential of the samples was monitored for 5 days to determine their stability. As shown in Tables S1 and S2, there was no significant increase or decrease in the surface charge of nanoparticles. The strong interaction between the nanoparticles and the plant extracts’ phytochemicals, such as -OH and -COOH, could be responsible for the negative charges. This surface adsorption is strongly associated with the stability of nanoparticles in water, thereby preventing aggregation in solution [48]. These high negative values can indicate long-term nanoparticle stability and comparable to other reports [36,49]. Anyway, a low polydispersity index (PDI) value (Tables S1 and S2), an indication of the monodispersity and quality of the nanoparticles, should be beneficial for this study as it influences the uptake, accumulation and, consequently, their biological outcome [36].

### 3.3. Plant Metabolites

**Figure 5:**
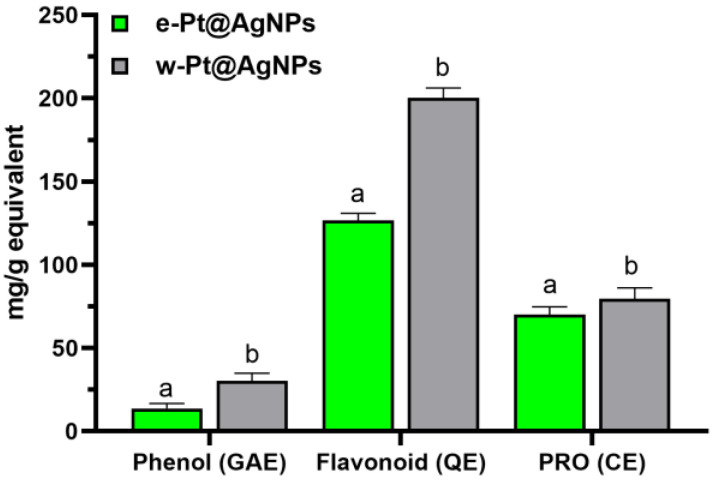
The polyphenolic contents were obtained after the synthesis of *w*-Pt@AgNPs and *e*-Pt@AgNPs. Values within the same histogram with different superscripts are significantly different (*p* < 0.05).

We examined how crucial certain plant metabolites (total phenolic content (TPC), total flavonoids content (TFC), and proanthocyanidins (PRO) content are in the synthesized *w*-Pt@AgNPs and *e*-Pt@AgNPs harnessed from *w*VM and *e*VM respectively, by way of measuring their content levels at the beginning of the synthesis (0 min) and after synthesis (60 min) to ascertain their probable mode of reaction. A previous study quantified the presence of total phenolic (39.07 + 0.01 mg GAE/g dry weight), flavonoids (171.58 + 0.02 mg QE/g dry weight), and proanthocyanin (34.55 + 1.10 mg CE/g dry weight) in aqueous *w*VM extract [41]. However, for the extract-synthesized nanoparticles (*w*-Pt@AgNPs and *e*-Pt@AgNPs, a reduction in the amount of TPC (30.28 ± 0.55 and 13.6 ± 0.1 mg GAE/g dry weight) and TFC (200.44 ± 5.87 and 126.6 ± 0.3 mg QE/g dry weight) was observed while the PRO content increased (79.57 ± 1.48 and 70.2 ± 0.6 mg CE/g dry weight) respectively, as shown in Figure 5.

### 3.4. Antioxidant Activity

**Figure 6:**
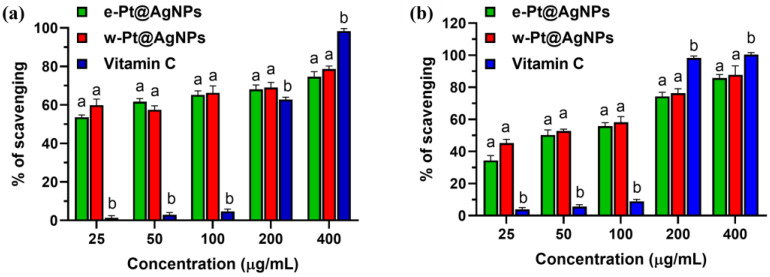
The inhibition of (**a**) ABTS and (**b**) DPPH radicals by *w*-Pt@AgNPs and *e*-Pt@AgNPs. Values are mean ± SD (*n* = 3). Mean separation by LSD. A set of bars (the same concentration) with different alphabets is significant.

In order to determine the antioxidant capacities of the synthesized *w*-Pt@AgNPs and *e*-Pt@AgNPs, three different assays were used, including ABTS, DPPH, and ferric reducing antioxidant power (FRAP) were carried out using vitamin C as a standard. The scavenging potential of *w*-Pt@AgNPs and *e*-Pt@AgNPs against ABTS and DPPH radicals are shown in Figure 6. It appears that both *w*-Pt@AgNPs and *e*-Pt@AgNPs nanoparticles exhibited dose-response tendencies for ABTS and DPPH scavenging activities. The results in Figure 6 show that *w*-Pt@AgNPs and *e*-Pt@AgNPs displayed higher inhibitory activity than Vitamin C at 25 to 100 µg/mL. The sharp increase in percentage inhibition of Vitamin C at 100 µg/mL to 200 µg/mL could be attributed to the drug concentration-effect relationship. As the Vitamin C concentration increases, the response rises to a maximum. At this point, the receptor is saturated [50]. Based on the ABTS and DPPH assays, we expressed the antioxidant activities as the 50% effective concentration (IC_50_) since it represents the concentration of the sample that can lead to a 50% reduction in the initial ABTS/DPPH concentration. The IC_50_ value also correlated with drug potency, i.e., the amount of drug necessary to produce the effect. Hence, the lower the IC_50_ value, the more potent the drug [51]. The low IC_50_ values of DPPH and ABTS radicals in this study (Table 2) suggest that *w*-Pt@AgNPs possess a superior antioxidant effect compared to *e*-Pt@AgNPs and vitamin C. Our findings agree with previous studies reporting nanoparticles possessing better scavenging potential than Vitamin C in terms of having a lower IC_50_ value [52,53]. Furthermore, our study revealed that both nanoparticles possessed reducing capacity, an attribute of antioxidant materials. Both *w*-Pt@AgNPs and *e*-Pt@AgNPs reducing potentials could be via donating a hydrogen atom to the ferric tripyridyltriazine complex, thus yielding ferrous tripyridyltriazine. Moreover, the *w*-Pt@AgNPs had a higher FRAP value of 81.54 mg GAE/g compared to the *e*-Pt@AgNPs value of 22.54 mg GAE/g (Table 2). It is noteworthy that the *w*-Pt@AgNPs exhibited an enhanced scavenging capability when compared with the *e*-Pt@AgNPs and ascorbic acid, as observed from the ABTS, DPPH, and FRAP radical scavenging assays. The scavenging potential displayed by the *w*-Pt@AgNPs could be due to the high surface area of the cubic-shaped nanoparticles [36,41], which permitted greater *w*VM phytochemical content stabilization for nanoparticles.

### 3.5. Cytotoxic Effect of Pt@AgNPs on Cancer Cells

**Figure 7:**
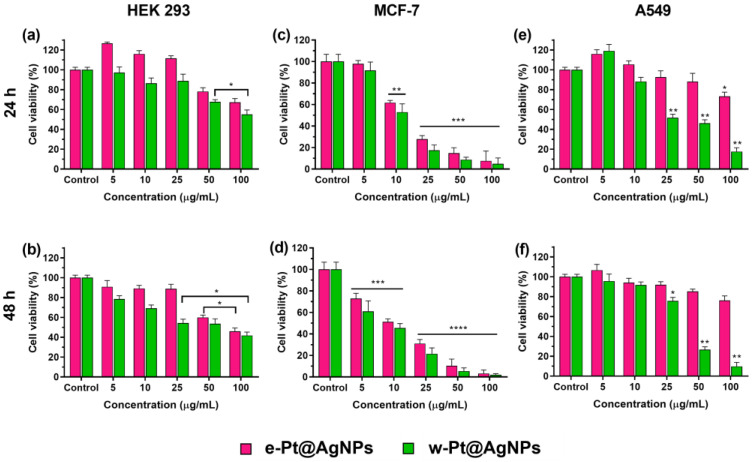
Cytotoxicity evaluation of *e*-Pt@AgNPs and *w*-Pt@AgNPs against (**a**,**b**) HEK 293, (**c**,**d**) MCF-7, (**e**,**f**) A549 cells incubated at 24 h and 48 h (*n* = 3). Data are expressed as a percentage of the untreated cells (always 100%) and represented as the mean ± standard deviation (SD). Statistical significance denoted as * p < 0.05, ** *p* < 0.01, *** *p* < 0.005, **** *p* < 0.001 are compared to untreated controls.

This study was aimed at assessing the therapeutic potential of ethanolic extract-stabilized *e*-Pt@AgNPs and aqueous extract-stabilized *w*-Pt@AgNPs in the inhibition of cancer cell proliferation. To determine the possible cytotoxic effects, we first evaluated their impact on the viability of normal cell line HEK 293 in vitro. Under various concentration ranges, the *e*-Pt@AgNPs and *w*-Pt@AgNPs responded to the HEK 293 cells in a dose-dependent manner (Figure 7a,b). At 24 h incubation, as high as 126% and 78% viability were recorded for *e*-Pt@AgNPs at 5 and 50 µg/mL, respectively, while *w*-Pt@AgNPs resulted in ~11.2% loss of viability at 25 µg/mL (Figure 7a). However, after 48 h incubation, a significant loss of viability was recorded in both Pt@AgNPs. Specifically, at 50 µg/mL, about 40.0% and 46.3% cell death was induced by both *e*-Pt@AgNPs and *w*-Pt@AgNPs, respectively, indicating that viability was lost as the incubation time increased (Figure 7b). A half-maximal inhibitory concentration (IC_50_) value of (33.1 and 18.5 µg/mL) at 24 h and (45.39 and 17.88 µg/mL) at 48 h were calculated for *e*-Pt@AgNPs and *w*-Pt@AgNPs, respectively. For both MCF-7 and A549 cancer cell lines, similar dose-dependent inhibition patterns were noticed. As shown in Figure 7c,d, both nanoparticles demonstrated an enhanced antiproliferative ability from 24 h to 48 h at 10–100 µg/mL dosage in MCF-7 cells. Although both nanoparticles showed cytotoxic potential, the *w*-Pt@AgNPs exhibited a greater cytotoxic effect compared to *e*-Pt@AgNPs and increased with dosage and incubation time. The use of phytochemical-stabilized bimetallic nanoparticles has displayed promising results in mitigating toxic effects and improving nanoparticle treatment efficiency [22,36,54]. The calculated IC_50_ values in Table 3. suggested that less dosage of *w*-Pt@AgNPs (17.5 and 13.7 µg/mL) would result in significantly higher cell death in MCF-7 cells compared to *e*-Pt@AgNPs (23.8 and 17.2 µg/mL).

In contrast, some differences in the dose-response dynamics of the cell viability in A549 cells were observed. Unlike the *w*-Pt@AgNPs, which showed significant cell death (53.7% and 82.4%) at 24 h and (73.4% and 90.2%) at 48 h when exposed to a 50–100 µg/mL concentration range, the *e*-Pt@AgNPs exhibited ~85.2% viability at 50 µg/mL concentration even after 48 h treatment (Figure 7e,f). The impact of the *e*-Pt@AgNPs tested on A549 cells only led to ~76.2% viability upon a higher concentration (100 µg/mL) after 48 h, as shown in Figure 7f. These findings were promising and thus prompted us to investigate whether apoptosis was the mechanism driving the loss of viability in both cancer cell types after exposure.

### 3.6. Exposure of Cells to e-Pt@AgNPs/w-Pt@AgNPs Enhances Cell Apoptosis

**Figure 8:**
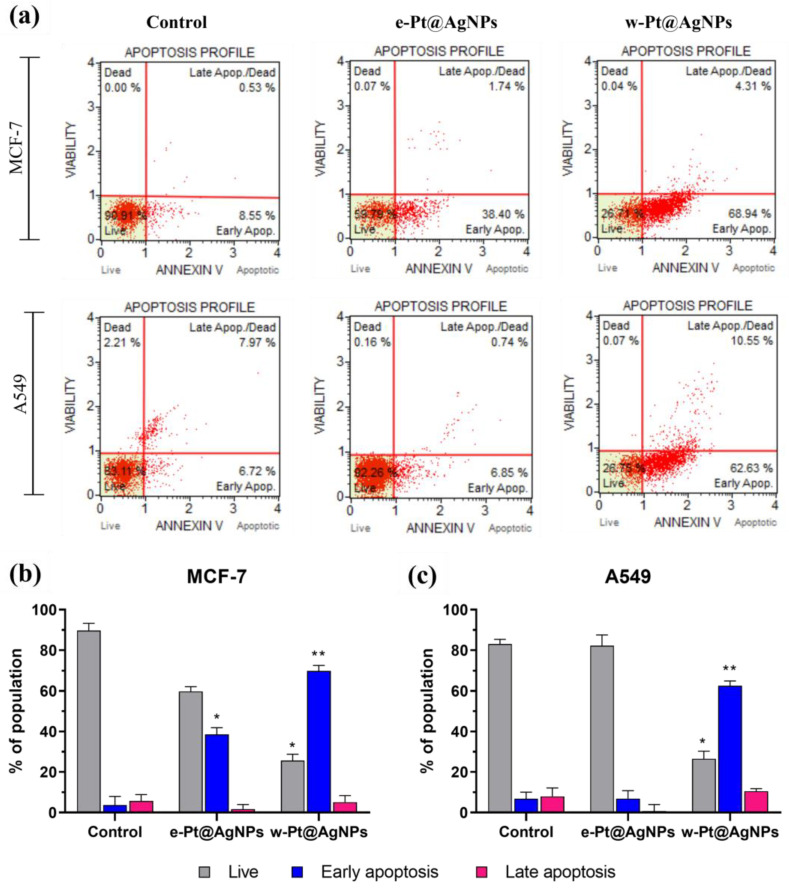
(**a**) Representative apoptotic profile plots of *e*-Pt@AgNPs and *w*-Pt@AgNPs on MCF-7 and A549 cells. Flow cytometric analysis showing the percentage of the cell population of (**b**) MCF-7 and (**c**) A549 cells following incubation with *e*-Pt@AgNPs and *w*-Pt@AgNPs for 24 h. Untreated cells are denoted as control. Data presented as mean ± SD with the differences * *p* < 0.05 and ** *p* < 0.01 considered significant.

The possible induction of apoptosis as the mechanism of cell death by the extract-synthesized *e*-Pt@AgNPs and *w*-Pt@AgNPs in cancer cells was investigated using flow cytometric analysis. Upon the rupture of the plasma membrane, phosphatidylserine (PS), an apoptosis-induction biomarker, can be translocated to the outer leaflet of the membrane, thereby enabling the determination of the various stages of cell health [20]. The apoptotic response of MCF-7 and A549 cells treated with *e*-Pt@AgNPs (IC_50_ = 23.8 and 100 µg/mL) and *w*-Pt@AgNPs (IC_50_ = 17.52 and 43.18 µg/mL), respectively for 24 h were analyzed with the MUSE Cell Analyzer after staining with Annexin V and Dead Cell reagent. The results obtained are shown in Figure 8a, where the percentage of MCF-7 cells undergoing apoptosis increased to 73.3% for *w*-Pt@AgNPs when compared with 40.1% for *e*-Pt@AgNPs. However, for A549 cells, the exposure of cells to *e*-Pt@AgNPs only led to 7.6%, while *w*-Pt@AgNPs induced 73.18% apoptosis. The difference in *w*-Pt@AgNPs treatment on both cells was that more cells in MCF-7 undergo early apoptosis than in A549 cells. Although both *e*-Pt@AgNPs and *w*-Pt@AgNPs induced apoptosis, the *w*-Pt@AgNPs were more efficient in inducing apoptosis on MCF-7 than on A549 cells at *p* < 0.01 (Figure 8b,c).

### 3.7. Cell Cycle Arrest

**Figure 9:**
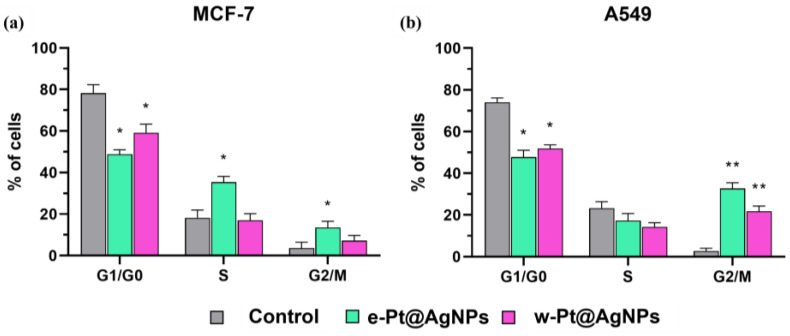
Cell cycle distribution plots of *e*-Pt@AgNPs and *w*-Pt@AgNPs after treatment on (**a**) MCF-7 and (**b**) A549 cancer cells at 24 h. Untreated cells are denoted as control. Data represented as mean ± SD from three independent experiments with differences * *p* < 0.05 and ** *p* < 0.01 considered significant.

The confirmation of the induction of apoptosis by the extract-synthesized *e*-Pt@AgNPs and *w*-Pt@AgNPs motivated the investigation of the cell cycle distributions occurring at different stages of mitosis. The ability of nanoparticles to inhibit cancer cell growth by cell cycle arrest at a specific checkpoint is the hallmark of many cytotoxic agents. The cells were treated with *e*-Pt@AgNPs (IC_50_ = 23.8 and 100 µg/mL) and *w*-Pt@AgNPs (IC_50_ = 17.52 and 43.18 µg/mL), respectively, for 24 h and then stained with MUSE cell cycle reagent. The different phases of cell division were analyzed by flow cytometry. In Figure 9a, the treatment of MCF-7 cells with *e*-Pt@AgNPs and *w*-Pt@AgNPs decreased the G0/G1 phase from 78.2% (control) to 48.8% and 59.1%, respectively. Consequently, the G2/M phase also increased in cell population from 3.6% to 13.5% and 7.2% for *e*-Pt@AgNPs and *w*-Pt@AgNPs. Although we noticed a decrease in the G0/G1 phase for both *e*-Pt@AgNPs and *w*-Pt@AgNPs, the result showed an accumulation of cells in the S phase for *e*-Pt@AgNPs (35.4%). Following the treatment of A549 cells with *e*-Pt@AgNPs, cells in the G2/M phase increased to 32.7%, while that of the *w*-Pt@AgNPs increased to 21.8% (Figure 9b). The increase in the percentage proportion of cells at the G2/M phase was accompanied by a reduction in the G0/G1 phase of the *e*-Pt@AgNPs (47.8%) and *w*-Pt@AgNPs (51.9%) when compared with the control cells (74.0%). The disruption of the G0/G1 suggested that both *e*-Pt@AgNPs and *w*-Pt@AgNPs arrest cells via the G2/M phase and are, therefore, responsible for the anticancer inhibition of growth.

## 4. Discussion

Many studies have highlighted the advantages of using plant extracts due to their eco-friendly, cost-effective, and sustainable means [36,55]. The abundance of bioactive constituents in the *w*VM presents an opportunity for the synthesis of a variety of noble-metal nanoparticles via a green-synthetic approach [41]. The reduction in Pt^2+^ and Ag^+^ with *w*VM and *e*VM extracts to form *w*-Pt@AgNPs, and *e*-Pt@AgNPs was achieved in a facile synthetic method. Despite the simultaneous mixing of the Ag^+^ and Pt^2+^ prior to the addition of the extracts, we anticipated a co-reduction process should occur during the formation of both nanoparticles. However, for *w*-Pt@AgNPs, it appears that the reduction in Ag^+^ took preference over that of Pt^2+^, judging by its structural architecture. Hence, we propose the mechanism and formation of the aqueous extract-synthesized *w*-Pt@AgNPs as suggested: The Ag^+^ seems to form cube-shaped Ag nanoparticles as template seeds first, followed by rapid deposition of Pt nanodot clusters onto its surfaces. Despite their miscibility gap, Ag can separate due to a lower surface energy (1.250 J m^−2^) compared to 2.475 J m^−2^ for Pt, consequently favoring AgNPs formation [56]. Moreso, the reduction potential of Ag^+^ (0.799 V) is much lower than that of Pt^2+^ (1.18 V). Therefore, the plant extract rich in potent reducing phytoconstituents (flavonoids, tannins, phenols) can quickly release electrons for the reduction in the Ag ions first before the Pt ions. Consequently, the observed morphological, size and zeta potential differences in both nanoparticles could be due to the effect of the type of extract solution used. With *e*VM extract, the reduction in metal ions and subsequent nucleation could significantly occur faster than with the *w*VM extract. The interaction between ethanol, a more polar solvent, and plant phytochemicals could remarkably cause a reduction in the particle size [57]. Moreover, a greater amount of charged ions from the *e*VM extract get adsorbed onto the surface of the *e*-Pt@AgNPs, thus leading to decreased zeta potential. As evidence, Hussain et al. investigated the synthesis of gold nanoparticles with different sizes using solvents of different polarities. Experimental results showed that with increasing solvent polarity, the zeta potential values of the nanoparticles decreases [58].

Several plant-derived compounds such as alkaloids, amines, coumarins, flavonoids, glycosides, phenols, proanthocyanins, steroids, and related metabolites are extensively explored in the drug and pharmaceutical industry [59,60]. Flavonoids, phenols, and proanthocyanins are some of the plant-derived compounds capable of donating electrons, chelating metals, and acting as quenchers of both singlet and triplet oxygen [42,61]. The shift in the FTIR peaks of the nanoparticles could be attributed to the interaction between bioactive compounds and metal ions during the formation. Our findings agree with our previous study, which depicted a reduction in the TFC and TPC levels of both Pt@AgNPs and PRO after their formation [36]. The noticed decrease could be ascribed to the electrophilic or nucleophilic nature of these plant-derived compounds by way of potentiating the chelating and reducing of transitional metals [62]. Previous studies have shown that plant extract-synthesized nanoparticles correlate well with antioxidant capacity using DPPH, ABTS, and FRAP assays [63,64]. The present results revealed that *w*-Pt@AgNPs and *e*-Pt@AgNPs demonstrated strong antioxidant activity compared to standard. Since the *w*-Pt@AgNPs and *e*-Pt@AgNPs have the potential to scavenge free radicals, therefore; it could be used to prepare suitable formulations for the safe and effective treatment of several diseases. Moreover, nanoparticles have become more desirable than synthetic antioxidants because they offer improved bioavailability and stability and enhance bioactive compounds delivery [64].

Through CellTitre Blue, Annexin V, and Dead experiments, we confirmed the enhanced killing effect of *w*-Pt@AgNPs on cancer cells compared to *e*-Pt@AgNPs at the same concentration. From our results, we could ascribe the enhanced cell death induced by the *w*-Pt@AgNPs on both MCF-7 and A549 cells to a number of factors; (1) the optical and structural properties of the large surface and contact areas [65], (2) greater antioxidant properties [22], and (3) the synergistic effect of Pt nanodots distribution on the atomically flat facets of the Ag cube template [66]. With the calculated IC_50_ values of (>100 and 43.2 µg/mL) at 24 h for both *e*-Pt@AgNPs and *w*-Pt@AgNPs in A549 cells, we concluded that a higher dosage possibly might be required to cause significant cell death. In addition, *w*-Pt@AgNPs demonstrated an enhanced apoptotic response in both cell lines compared to *e*-Pt@AgNPs. Unlike *w*-Pt@AgNPs, the *e*-Pt@AgNPs displayed an alloyed orientation with reduced surface area. We suspect that the lower antioxidant potential and confinement of ions in a tightly-fused alloyed configuration could have limited its killing effect on A549 cells [36]. In addition, the resilience of non-small cell lung cells such as A549 to drug/nanoparticles is well documented [66,67]. The resistance of A549 cells to drug/nanoparticle uptake and transport has been predominantly attributed to their high overexpression of the intracellular lung-resistant protein, which confers cytoprotection onto the cells [68]. Moreover, the mechanism of cell uptake, the diffusion pattern of either Pt nanodot or Ag ions, and the physicochemical properties of the *w*-Pt@AgNPs could also contribute to the selective nature and cytotoxic responses observed. These data suggested that the phytochemical-capped *e*-Pt@AgNPs and *w*-Pt@AgNPs can induce programmed cell death via an apoptotic mechanism.

With bimetallic nanoparticles, the spatial arrangement of metal atoms in the structural architecture may play a key role in regulating their cell cycle arrest. For example, alloyed Pt@Cu nanoparticles showed fragmented DNA upon treatment in cervical cells via sub-G0 phase cell arrest [69]. An induction of apoptosis resulting from the interruption of cell cycle progression by *w*-Pt@AgNPs in tumor cells through a triggered G2/M cell cycle phase could be pivotal in regulating the therapeutic outcome of extract synthesized nanoparticles. These findings are consistent with earlier reports on the induction of the G2/M phase by AgNPs and Au@Pt nanoparticles in glioblastoma multiforme (GBM) and cervical cells [70,71]. Several noble metal-based nanoparticles have been reported to arrest the cell cycle at different stages. The induction of apoptosis via the G2/M phase arrest by platinum nanoparticles in human cervical cells [72], as well as the induction of the G1 phase by gold nanoparticles in multiple myeloma cells [73], provided invaluable information regarding the dynamics of nanoparticle-cell cycle arrest. Hence, our results indicated that *w*-Pt@AgNPs are not just efficient in killing breast cancer cells but also the more resilient lung cancer cells. In the future, it will be interesting to study the molecular mechanism and optimize the *w*-Pt@AgNPs for target-specific therapies.

## 5. Conclusions

Bimetallic platinum nanodots-decorated silver nanocubes (*w*-Pt@AgNPs) were successfully synthesized using a facile, eco-friendly, and inexpensive *V. mespilifolia* aqueous extract. The *w*-Pt@AgNPs resulted from the effective stabilization, capping, and reducing properties of the phytochemicals contained in the aqueous extract. The *w*-Pt@AgNPs showed a greater scavenging activity on free radicals, especially ABTS and DPPH, when compared with *e*-Pt@AgNPs and ascorbic acid. In addition, the *w*-Pt@AgNPs exhibited improved anticancer activities in a dose and time-dependent manner via an apoptotic mechanism of cell death. The combination of antioxidant and anticancer properties proved efficient in inhibiting breast cancer cells as well as the more resistant lung cancer cells. These findings suggest that bimetallic *w*-Pt@AgNPs with highly exposed Pt surfaces could be a promising nanoplatform for anticancer therapy.

## Figures and Tables

**Table 1:** Mass spectrometric data of key compounds found in the extract of *w*VM.

RT (min)	Compounds	Molecular Formula	Molecular Weight	Main Fragments (*m*/*z*)	Structure
0.83	N6-Methyl-2″-deoxyadenosine	C_11_H_15_N_5_O_3_	265.117	248, 230, 116	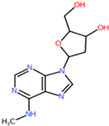
0.89	Proline	C_5_H_9_NO_2_	115.063	163, 116, 70	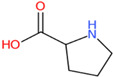
1.01	Vigabatrin	C_6_H_11_NO_2_	129.079	343, 193, 130	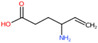
1.15	4-Guanidino-1-butanol	C_5_H_13_N_3_O	131.106	343, 199, 146, 130	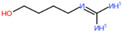
1.21	4-Guanidinobutanoate	C_5_H_11_N_3_O_2_	145.085	329, 146, 130, 112	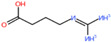
1.87	Phenylpyruvate	C_9_H_8_O_3_	164.047	343, 165, 123	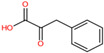
3.54	Bis-D-fructose2″,1:2,1″-dianhydride	C_12_H_20_O_10_	342.106	487, 325, 163	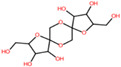
4.71	epsilon-Caprolactam	C_6_H_11_NO	113.084	343, 295, 196, 114	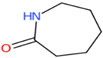
5.18	2-Hydroxy-7-hydroxymethylchromene-2-carboxylate	C_11_H_10_O_5_	222.053	343, 223, 161	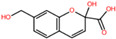

**Table 2:** FRAP value and IC_50_ from the *w*-Pt@AgNPs, *e*-Pt@AgNPs, and Standard Drug.

Samples/Standard	ABTS IC50 (μg/mL)	DPPH IC50 (μg/mL)	FRAP (mg GAE/g)
*e*-Pt@AgNPs	21.50 ± 1.63 ^a^	19.92 ± 0.05 ^a^	44.54 ± 1.62 ^a^
*w*-Pt@AgNPs	14.52 ± 2.41 ^b^	14.22 ± 0.19 ^b^	81.54 ± 1.30 ^b^
Ascorbic acid	191.03 ± 0.68 ^c^	126.83 ± 0.58 ^c^	-

Abbreviations: IC_50_, the concentration (mg/mL) required to scavenge/inhibit 50% of the radical.

**Table 3:** The calculated IC_50_ values (µg/mL) for *e*-Pt@AgNPs and *w*-Pt@AgNPs in HEK 293, MCF-7, and A549 cells incubated for 24 h and 48 h.

		24 h			48 h	
Nanoparticles	HEK 293	MCF-7	A549	HEK 293	MCF-7	A549
*e*-Pt@AgNPs	33.06	23.80	>100	45.39	17.23	32.81
*w*-Pt@AgNPs	18.50	17.52	43.18	17.88	13.68	20.96

## Data Availability

The data presented in this study are available in this article or supplementary material.

## Supplementary Materials

The following supporting information can be downloaded at: https://www.mdpi.com/article/10.3390/pharmaceutics14112541/s1, Figure S1: Particle size distribution showing the average diameter of *w*-Pt@AgNPs and *e*-Pt@AgNPs Figure S2; Representative EDS profile of *e*-Pt@AgNPs confirming the presence of Ag and Pt metals Figure S3: Selected area electron diffraction (SAED) pattern of *e*-Pt@AgNPs; Table S1: Zeta-potential and polydispersity index (PDI) of *w*-Pt@AgNPs (Mean ± SD; *n* = 3) Table S2. Zeta-potential and polydispersity index (PDI) of *e*-Pt@AgNPs (Mean ± SD; *n* = 3).
