# Supplementary material for: Phytochemical-Stabilized Platinum-Decorated Silver Nanocubes INHIBIT Adenocarcinoma Cells and Enhance Antioxidant Effects by Promoting Apoptosis via Cell Cycle Arrest
Source: Pharmaceutics. 2022 Nov 21;14(11):2541. doi: 10.3390/pharmaceutics14112541 (PMC9693179; doi:10.3390/pharmaceutics14112541)
Supplement: Supplementary file 1 [file pharmaceutics-14-02541-s001.zip › pharmaceutics-2007774-supplementary.pdf]

Supplementary Information

Phytochemical-stabilized platinum-decorated silver nanocubes inhibit adenocarcinoma cells and enhance antioxidant effects by promoting apoptosis via cell cycle arrest

Adewale O. Oladipo [1*], Jeremiah O. Unuofin [1], Sogolo L. Lebelo [1], Titus A.M. Msagati [2]

[1]Department of Life and Consumer Sciences, College of Agriculture and Environmental Sciences, University of South Africa, Private Bag X06 Florida 1710, South Africa

[2]Institute for Nanotechnology and Water Sustainability (iNanoWS), College of Science, Engineering and Technology, University of South Africa, Private Bag X06 Florida 1710, South Africa

Author for correspondence: oladiao@unisa.ac.za

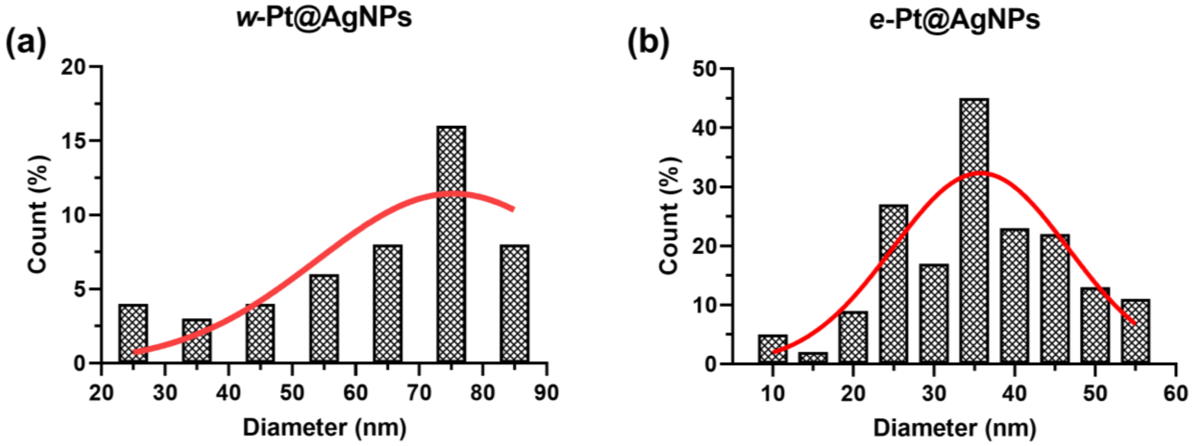

Figure S1. Particle size distribution showing the average diameter of *w*-Pt@AgNPs and *e*-Pt@AgNPs.

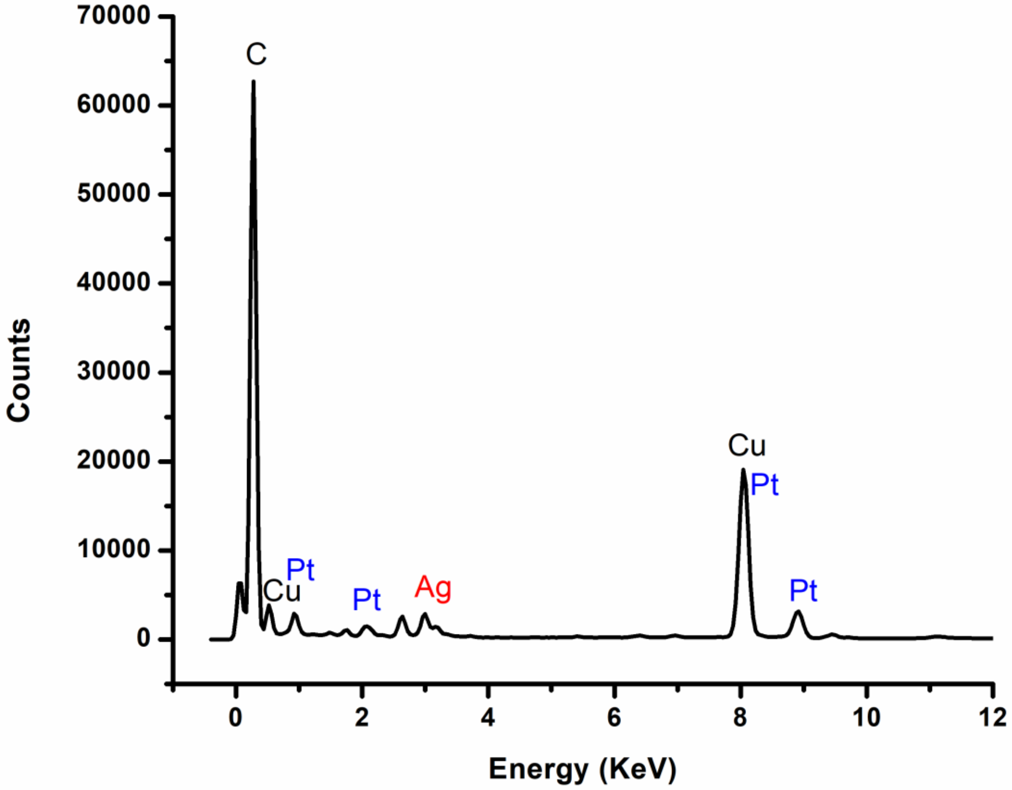

Figure S2. Representative EDS profile of *e*-Pt@AgNPs confirming the presence of Ag and Pt metals.

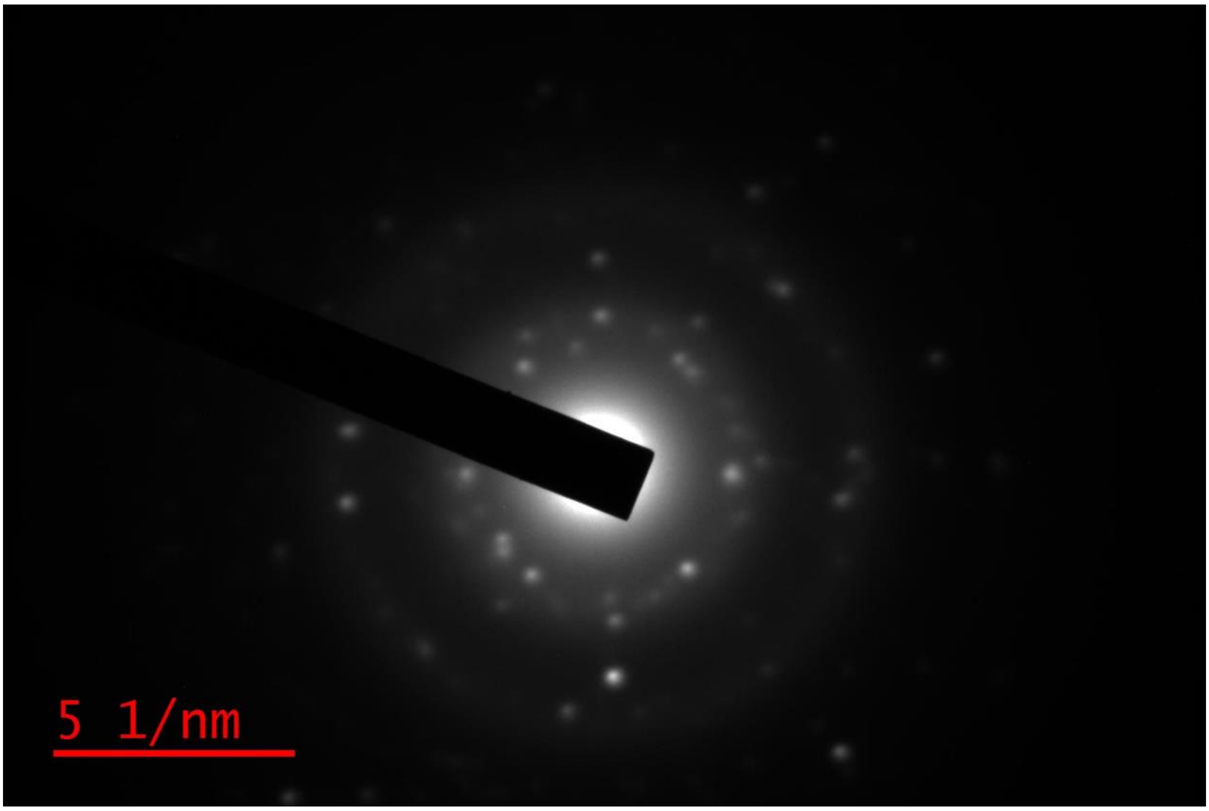

Figure S3. Selected area electron diffraction (SAED) pattern of *e*-Pt@AgNPs.

Table S1. Zeta-potential and polydispersity index (PDI) of *w*-Pt@AgNPs (Mean ±SD; n=3).

Days	Particle size (nm)	PDI	Zeta potential (mV)
0	84.2 ± 1.39	0.220	-30.3 ± 2.81
1	85.3 ± 0.45	0.214	-30.2 ± 1.66
2	85.1 ± 2.08	0.184	-30.5 ± 2.13
3	85.4 ± 1.91	0.209	-30.4 ± 2.53
4	85.7 ± 3.12	0.216	-30.3 ± 4.31
5	86.0 ± 1.87	0.197	-30.6 ± 3.82

Table S2. Zeta-potential and polydispersity index (PDI) of *e*-Pt@AgNPs (Mean ±SD; n=3).

Days	Particle size (nm)	PDI	Zeta potential (mV)
0	39.4 ± 2.01	0.109	-25.0 ± 2.11
1	39.3 ± 1.34	0.213	-24.8 ± 3.02
2	39.7 ± 3.12	0.401	-25.3 ± 1.06
3	39.8 ± 2.63	0.311	-25.4 ± 1.66
4	40.1 ± 4.07	0.342	-25.3 ± 1.31
5	40.4 ± 5.42	0.308	-26.0 ± 2.17
